# Nomogram for Predicting Efficacy and Prognosis After Chemotherapy for Advanced NSCLC

**DOI:** 10.1111/crj.13815

**Published:** 2024-08-08

**Authors:** Jiaying Gao, Yandong Nan, Gang Liu, Shihong Zhao, Huanqing Xiong, Yifeng Wang, Faguang Jin

**Affiliations:** ^1^ Department of Respiration, Tangdu Hospital Air Force Medical University Xi'an Shaanxi China; ^2^ Department of Respiration Shaanxi University of Chinese Medicine Xianyang Shaanxi China

**Keywords:** 7‐AAbs, chemotherapy, NLR, PLR, prognosis

## Abstract

**Purpose:**

One major issue is the therapeutic effect following chemotherapy for non–small cell lung cancer (NSCLC). Although numerous risk factors have been identified and novel therapies have been developed, improving patient overall survival (OS) remains a crucial postoperative issue. This study aimed to develop a nomogram for accurately predicting the OS of patients with Stage III–IV NSCLC treated with chemotherapy.

**Methods:**

The Department of Respiration at Tangdu Hospital, Air Force Medical University, prospectively collected data on 321 patients between January 2018 and December 2023. A week before treatment, the platelet‐to‐lymphocyte ratio (PLR), the neutrophil‐to‐lymphocyte ratio (NLR), and seven autoantibodies were measured using Youden's index, which was obtained using the ROC curve. The formula was used to compute the values of PLR and NLR. After using multifactor Cox regression analysis to identify risk factors, a nomogram was produced regarding the therapeutic effect following chemotherapy. The performance of the nomogram was assessed using a bootstrapped‐concordance index and calibration plots.

**Result:**

It was determined that NLR, sex‐determining region Y‐box 2 (SOX2), adenosine triphosphate binding RNA deconjugase 4–5 (GBU4‐5), and MAGE family member A1 (MAGEA1) were significantly associated factors that could be combined to accurately predict the therapeutic effect following chemotherapy. Utilizing these risk indicators, we were able to develop a nomogram that predicted the patients' survival at 1, 3, and 5 years. At 3 years, the area under the curve representing the expected survival probability was 0.762 (95% confidence interval 0.66–0.87). With a bootstrapped‐concordance index of 0.762, the nomogram demonstrated good calibration.

**Conclusions:**

Our nomogram proved to be a valuable instrument in accurately predicting the overall survival of patients.

## Graphical abstract

The nomogram was built on data of 321 patients with Stage III–IV NSCLC, and NLR, SOX2, GBU4‐5, and MAGEA1 were identified as significantly associated factors that could be combined for accurate prediction of therapeutic effect after chemotherapy. The model demonstrated excellent predictive performance.

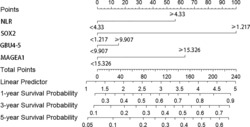

## 1 Introduction

Both the supervising physician and patients with non–small cell lung cancer (NSCLC) have significant challenges in achieving a therapeutic outcome. Patients with advanced NSCLC are currently treated primarily with chemotherapy [1]. The clinical evaluation of chemotherapy efficacy is still primarily conducted using the Response Evaluation Criteria in Solid Tumors (RECIST1.1) [2], which is primarily based on the clinical experience of the doctors and is insufficiently objective. Therefore, more objective indexes can be used to assess chemotherapy efficacy. We advocate for more extensive research to be conducted on the variations in peripheral serological indicators and levels of the seven autoantibodies (7‐AAbs) in advanced lung cancer patients before treatment. This research could help determine whether these indicators can serve as biomarkers for evaluating the effectiveness of chemotherapy in advanced NSCLC. Such findings would hold significant importance in enhancing the survival rates of lung cancer patients and assessing therapeutic efficacy.

A nomogram is a visual representation of a predictive model that combines multiple risk factors to enable accurate predictions. They are utilized as predictive tools in various diseases, including lung cancer, to aid in precise prognostication [3, 4, 5, 6]. We believed that employing a nomogram for predicting the therapeutic outcome following chemotherapy in lung cancer could be beneficial. The aim of this research was to create a nomogram using data from the Department of Respiration at Tangdu Hospital, Air Force Medical University, to enhance the accuracy of predicting the therapeutic effect after chemotherapy for NSCLC.

## 2 Materials and Methods

### 2.1 Patients

We prospectively collected data of 1483 consecutive patients with advanced non‐small cell lung cancer from the Department of Respiration, Tangdu Hospital, Air Force Medical University, between January 2018 and December 2023 to investigate the therapeutic effect after chemotherapy. We excluded 1162 patients who missed visits or incomplete examination results and included the remaining 321 patients. This study was approved by the Ethics Committee of Tangdu Hospital. Informed consent was obtained from all patients.

### 2.2 Statistical Analysis

Overall survival (OS) was defined as from the day of treatment to the date of death or last follow‐up.

All *p* values were two‐sided, and the risk factors with *p* values < 0.10 in univariate analysis were included in a multivariate analysis. The optimal cutoff values for platelet‐to‐lymphocyte ratio (PLR), neutrophil‐to‐lymphocyte ratio (NLR), and each autoantibody were determined through ROC curves, with selection based on Youden's index. Identifying independent risk factors was achieved through multivariate logistic regression analysis, employing a stepwise method to pinpoint the most precise combination of factors for predicting therapeutic effectiveness. Based on the system proposed by the WHO of Response Evaluation Criteria in Solid Tumors (RECIST1.1), response was classified into three categories: Grade A, complete response and partial response; Grade B, progressive disease; and Grade C, stable disease. We defined all grades as outcomes in this study. A nomogram for OS was constructed using the results from the multivariate logistic regression model. The nomogram's performance was assessed using a concordance index and calibration plots utilizing bootstrap samples. The concordance index served as a numerical measure of discriminative ability, whereas the calibration plots offered graphic evaluations of predictive accuracy by comparing observed probabilities with nomogram‐predicted probabilities. All statistical analyses were conducted using the R statistical software, and the nomogram was developed using the “rms” package [7].

## 3 Results

### 3.1 Patient Characteristics

A total of 321 patients were included in the analysis. The clinical characteristics of the patients are shown in Table 1. Univariate and multivariate analysis statistical significances are shown in Table 2. The values of NLR, SOX2, GBU4‐5, and MAGEA1 in multivariate logistic regression analysis are 0.02, < 0.001, 0.027, and 0.004, respectively. The platelet, lymphocyte, and neutrophil were measured preoperatively.

**TABLE 1:** Patient characteristics.

Actor	*n*	%
Sex		
Male	212	66.0
Female	109	34.0
Age		
< 60	135	42.0
≥ 60	186	58.0
Smoking history		
Never	142	44.2
Former	39	12.1
Current	140	43.6
Pathological type		
Squamous carcinoma	132	41.1
Adenocarcinoma	189	58.9
ECOG		
0	82	25.5
1	188	58.6
2	51	15.9
Clinical stage		
III	109 (45.2)	45.2
IV	132 (54.7)	54.7
NLR		
≥ 4.33	101	31.5
< 4.33	220	68.5
PLR		
≥ 65.41	309	96.3
< 65.41	12	3.7
P53 (U·L^−1^)		
≥ 1.457	237	73.8
< 1.457	84	26.2
PGP9.5 (U·L^−1^)		
≥ 3.08	167	52.0
< 3.08	154	48.0
SOX2 (U·L^−1^)		
≥ 1.217	169	52.7
< 1.217	152	47.3
GAGE7 (U·L^−1^)		
≥ 1.939	228	71.0
< 1.939	93	29.0
GBU4‐5 (U·L^−1^)		
≥ 9.907	57	17.8
< 9.907	264	82.2
MAGEA1 (U·L^−1^)		
≥ 15.326	41	12.8
< 15.326	280	87.2
CAGE (U·L^−1^)		
≥ 0.368	57	17.8
< 0.368	264	82.6

**TABLE 2:** Factors associated with survival rate of advanced NSCLC patients.

Factor		Univariate analysis			Multivariate analysis		
		OR	95% CI	*p*	OR	95% CI	*p*
Sex	(Male/female)	1.03	0.69–1.55	0.077			
Age	(≥ 60/ <60)	0.51	0.68–2.20	0.508			
Smoking history	(Never/former/current)	1.35	0.39–4.72	0.036			
Pathological type	(Squamous carcinoma/adenocarcinoma)	0.59	0.01–68.15	0.128			
ECOG	(0/1/2)	0.59	0.01–94.99	0.14			
Clinical stage	(III/IV)	0.00	0.00–3.31	0.085			
NLR	(≥ 4.33/ <4.33)	0.80	0.16–4.07	0.041	0.77	0.61–0.96	0.020
PLR	(≥ 65.41/< 65.41)	6.12	0.00–27.78	0.037			
P53	(≥ 1.457/< 1.457)	0.02	0.66–1.56	0.348			
PGP9.5	(≥ 3.08/< 3.08)	2.62	0.00–32.63	0.22			
SOX2	(≥ 1.217/< 1.217)	0.00	0.00–82.50	< 0.001	0.26	0.15–0.44	< 0.001
GAGE7	(≥ 1.939/< 1.939)	0.30	0.00–1.21	0.522			
GBU4‐5	(≥ 9.907/< 9.907)	0.27	0.17–0.44	< 0.001	2.15	1.09–4.21	0.027
MAGEA1	(≥ 15.326/< 15.326)	0.46	0.00–7.11	0.021	0.41	0.23–0.75	0.004
CAGE	(≥ 0.368/< 0.368)	0.67	0.26–1.72	0.554			

### 3.2 Nomogram for Survival

**FIGURE 1:**
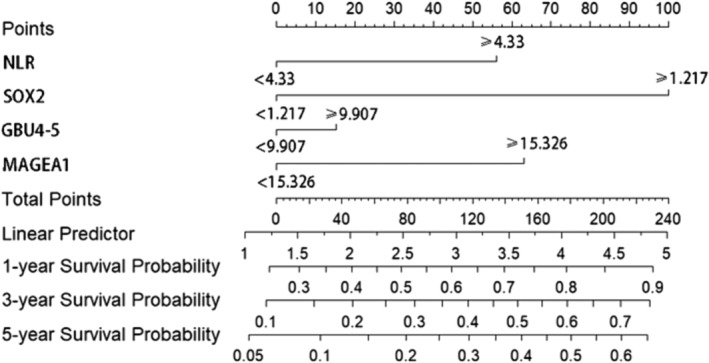
Survival nomogram for 1‐, 3‐, and 5‐year overall survival (OS) in advanced NSCLC patients.

**FIGURE 2:**
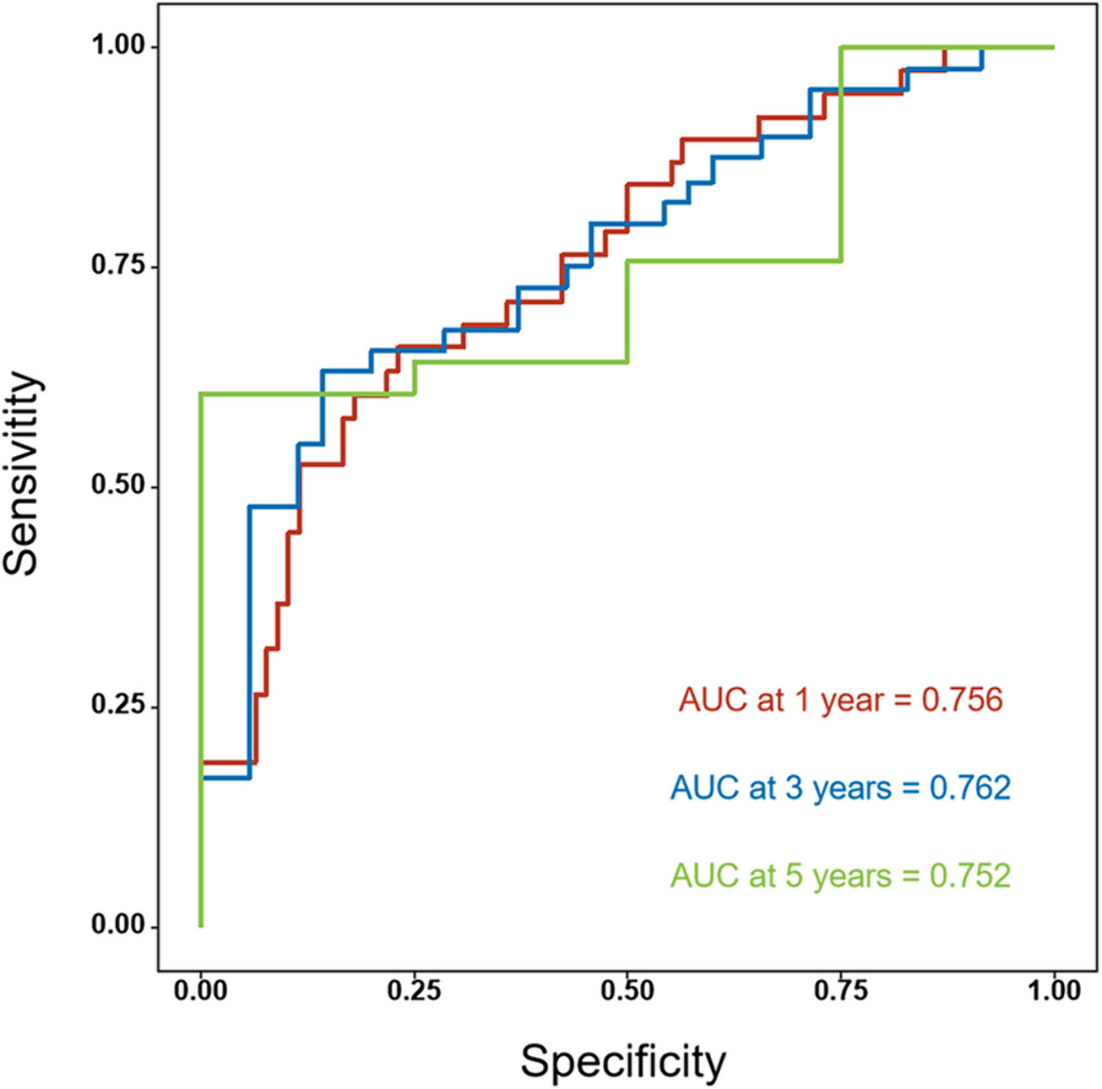
Receiver operating characteristic curve for the prediction model area under the curve.

**FIGURE 3:**
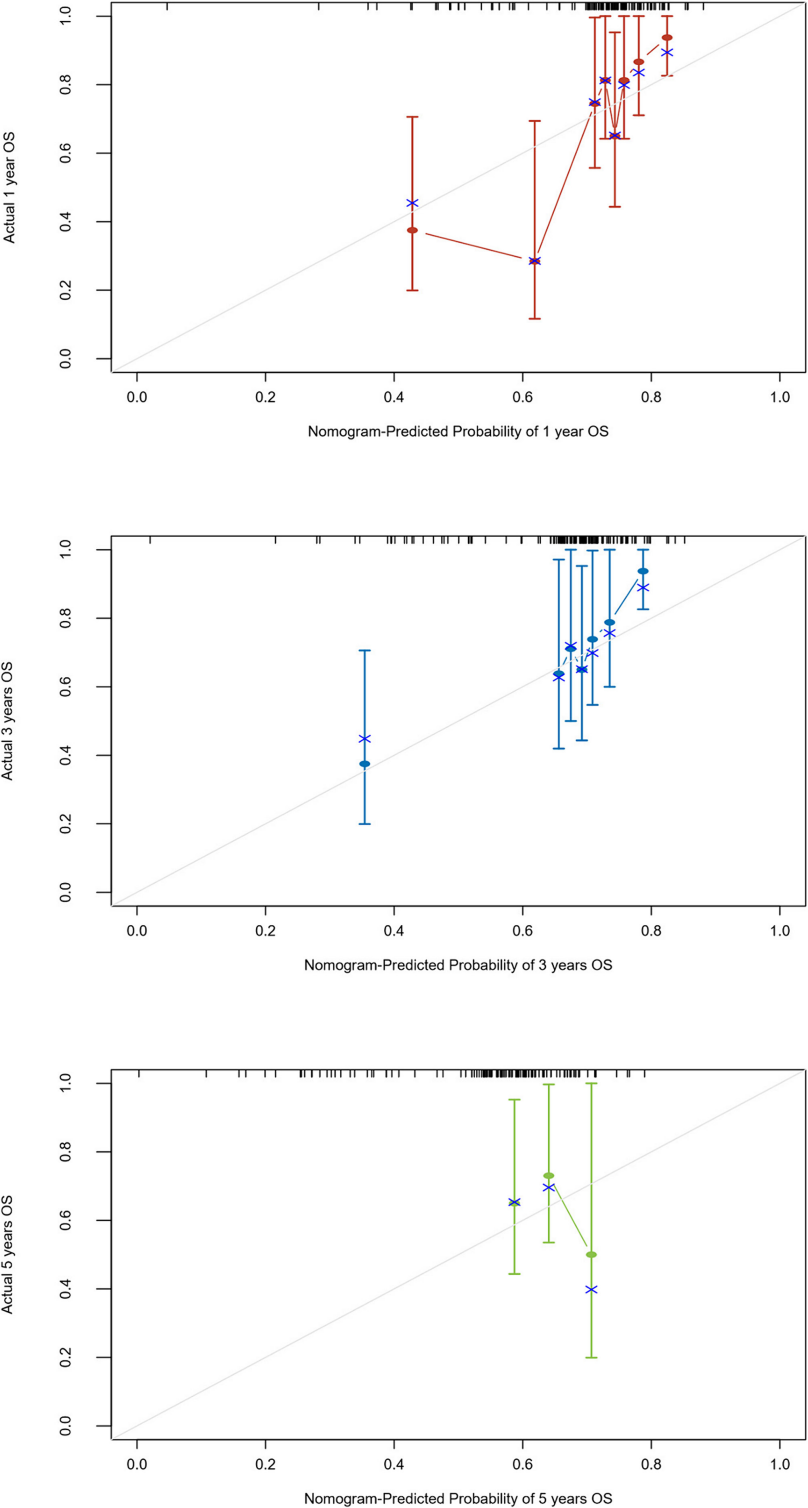
Calibration curves (A–C) of the nomogram for overall survival (OS) at 1, 3, and 5 years. x‐axis is the nomogram predicted probability of survival, and y‐axis is the actual probability of survival.

We examined the correlation between clinical factors and OS through univariate analysis. Notably, smoking history, NLR, PLR, SOX2, GBU4‐5, and MAGEA1 exhibited significant associations with the survival rate of advanced NSCLC patients (Figure 1). Employing multivariate logistic regression with a stepwise approach, we identified NLR, SOX2, GBU4‐5, and MAGEA1 as independent risk factors. Creating a nomogram for OS using these factors, we achieved an area under the curve (AUC) of 0.756 (95% CI 0.66–0.85), 0.762 (95% CI 0.66–0.87), and 0.752 (95% CI 0.53–0.97) for 1, 3, and 5 years, respectively (Figure 2). The nomogram demonstrated a bootstrapped‐concordance index of 0.7 and exhibited good calibration (Figure 3).

## 4 Discussion

In this research, we developed a nomogram to predict the OS of Stage III–IV NSCLC patients following chemotherapy [8, 9]. The usefulness of nomograms for cancer has been reported [10] with the advantage of accurate prediction OS in patients. Prior studies have delineated numerous peripheral serological markers, such as PLR and NLR, acknowledged as predictors of various cancers, including NSCLC, and capable of evaluating antitumor immune responses in patients [11, 12]. In this research, we identified sex, smoking history, clinical stage, NLR, PLR, SOX2, GBU4‐5, and MAGEA1 as potential predictors for evaluating the therapeutic effect post‐chemotherapy. Notably, the most impactful combination comprised NLR, SOX2, GBU4‐5, and MAGEA1. Each factor demonstrated discriminative ability with bootstrapped‐concordance indexes of 0.62, 0.57, 0.62, and 0.54, respectively. Consequently, we developed a highly discriminative nomogram with a concordance index of 0.7.

Innate immunity, characterized by the presence of inflammation, is a prevalent feature within the microenvironment of tumor tissues [13]. The extent of inflammation can be indicated by the levels of neutrophils, lymphocytes, platelets, and acute phase proteins [14], which are simple and easy to measure in clinical practice. In a retrospective study conducted by Shaverdian et al., a total of 149 NSCLC patients treated with stereotactic radiotherapy in Stage I were included in the study, and the results showed that NLR and PLR could be used as predictors of failure after stereotactic therapy [15]. Qu et al. included 389 patients who received concurrent radiotherapy to explore the prognostic value of PLR and NLR in their predictions. The results showed that the higher the NLR and PLR values, the worse the median OS (NLR: 14.13 m vs. 23.8 m, *p* < 0.001; PLR: 15.49 m vs. 22.04 m, *p* < 0.001) [16]. Currently, relevant studies have confirmed that peripheral serological indicators can be used as markers for predicting tumor prognosis [17, 18, 19, 20, 21], but they are still highly controversial and therefore not widely promoted. In this study, we assessed the predictive value of PLR and NLR, serologic inflammatory indexes, for the survival of patients with Stage III–IV NSCLC who underwent chemotherapy.

The tumor‐associated autoantibodies (TAAbs) have been studied as biomarkers in malignant tumors since early times, but they have been studied primarily as diagnostic markers [22], with little reference to prognosis [23, 24, 25, 26, 27]. In 2008, a study by Chapman et al. showed that the TAAb combination of P53, c‐myc, HER2, NY‐ESO‐1, CAGE, MUC1, and GBU4‐5 was positive in the peripheral blood samples of 76% of advanced lung cancer patients [28]. In 2008, Zhou et al. found the combination of TAAbs (P53, GAGE7, PGP9.5, CAGE, MAGEA1, SOX 2, and GBU4‐5) can be applied to the diagnosis of the malignancy of ground‐glass nodules [29]. Given the challenges associated with obtaining tissues in clinical settings, there is a growing interest among researchers in exploring noninvasive specimens, such as peripheral blood, for prognostic biomarkers. TAAbs, closely linked to the immune system, hold promise as prognostic predictors and are gaining attention for their potential value in forecasting outcomes.

In contrast, our focus was on prospectively collecting data on the combination of serologic markers and 7‐AAbs, investigating multiple factors potentially associated with OS, and conducting thorough analysis. Subsequently, we developed a nomogram by identifying the most effective combination for accurately predicting OS. Our nomogram offers physicians precise probabilities of OS after chemotherapy for advanced NSCLC. It incorporates clinical findings, serologic markers, and 7‐AAbs, which can be readily utilized just before chemotherapy. Identifying patients with lower survival probabilities post‐chemotherapy using the nomogram prompts careful monitoring during treatment or consideration of treatment modifications. The valuable insights from our nomogram empower clinicians to adjust treatment plans promptly before radiological evidence of progression. Notably, in 2009, Ohue et al. reported that patients with advanced NSCLC exhibiting low expression for the autoantibody combinations of NY‐ESO‐1 and XAGE1 experienced significant prolongation of PFS and OS after immunotherapy compared to those with high expression [30]. The present data contribute to enhancing the research significance of NLR, PLR, and 7‐AAbs in predicting the prognosis of advanced NSCLC patients. This provides a foundation for utilizing NLR, PLR, and 7‐AAbs in clinical predictions for advanced NSCLC prognosis. Our nomogram indicates that elevated NLR, SOX2, GBU4‐5, and MAGEA1 are associated with increased risk and lower OS.

Our study's strength lies in its use of prospectively collected data from previous studies focused on OS following chemotherapy for advanced NSCLC. We conducted a comprehensive investigation into multiple factors potentially associated with OS after chemotherapy for advanced NSCLC. Nevertheless, our study has certain limitations. It is a single‐center study with a small and non‐representative sample size, potentially leading to inaccurate results. Moreover, the applicability of the nomogram developed in this study to all clinicians is uncertain, as all participating institutions were in China, and the nomogram's performance was not evaluated in another cohort. Additionally, our inclusion of only common serologic indicators, such as platelets, neutrophils, and lymphocytes, may not fully reflect the patient's systemic status, and individual variations in nutritional status, comorbidities, and other factors, such as thoracic surgery [31], could impact the accuracy of our conclusions. Lastly, there are no exact optimal cutoff values for NLR and PLR.

## 5 Conclusion

Our nomogram demonstrates utility in predicting the probability of survival at 3 years, exhibiting strong predictive accuracy. Its application could facilitate the assessment of efficacy and prognosis following chemotherapy for advanced NSCLC.

## Author Contributions

Conception and design: Jiaying Gao, Yandong Nan, and Gang Liu. Administrative support: Faguang Jin. Provision of study materials or patients: Yifeng Wang. Collection and assembly of data: Jiaying Gao, Yifeng Wang, and Huanqing Xiong. Data analysis and interpretation: All authors. Manuscript writing: Jiaying Gao, Huanqing Xiong, and Shihong Zhao. Final approval of manuscript: All authors.

## Ethics Statement

This study was reviewed by the hospital Ethics Committee.

## Conflicts of Interest

The authors declare no conflicts of interest.

## Data Availability

The data that support the findings of this study are available from the corresponding author upon reasonable request.
